# Potential contribution of age-related and methodological factors to limited reproducibility in autism spectrum disorder blood miRNA biomarker studies: an exploratory meta-analysis

**DOI:** 10.1038/s41598-026-51487-x

**Published:** 2026-05-02

**Authors:** Kwanghwan Lim, Heejeong Shin, Seung-Nam Kim

**Affiliations:** 1 College of Korean Medicine, Dongguk University, Goyang, South Korea; 2 Barun Kyunghee Korean Medicine Clinic, Seoul, South Korea

**Keywords:** Autism spectrum disorder, MicroRNA, Meta-analysis, Methodological heterogeneity, Cross-validation, Blood biomarkers, Biomarkers, Diseases, Genetics, Medical research

## Abstract

Cross-study inconsistencies in autism spectrum disorder (ASD) blood microRNA biomarker studies suggest that methodological heterogeneity may substantially limit reproducibility. We conducted an exploratory meta-analysis of publicly available ASD blood miRNA datasets from the Gene Expression Omnibus, applying rigorous inclusion criteria and standardized analytical protocols. Three datasets were included (GSE89596, GSE67979, GSE222046) comprising 614 miRNAs across 90 participants (45 ASD, 45 controls). Random-effects meta-analysis was performed using Hedges’ g effect sizes, with comprehensive heterogeneity assessment and leave-one-dataset-out cross-validation. No miRNAs survived multiple testing correction (Benjamini-Hochberg FDR < 0.05), though seven candidate signals showed consistent evidence with unadjusted *p* < 0.01 and large effect sizes. These candidates demonstrated near-zero between-study heterogeneity and consistent directionality across validation analyses. Potential age-related and platform-related differences were observed, with near-zero correlation between adult and pediatric effect sizes (Kendall’s τ = -0.022); however, these two sources of variability were fully confounded in the available data and could not be separated. Some miRNAs exhibited extreme between-study variability (I² > 80%), indicating substantial methodological differences. Cross-validation revealed that excluding the single adult dataset reduced sign consistency from 89.9% to 68.9%. Our findings suggest that age-related and methodological factors, including technical platform differences, may contribute to limited reproducibility in ASD blood miRNA research, and that blood-derived signals should be interpreted as potentially reflecting peripheral physiological states rather than central disease mechanisms. A supplementary cross-tissue analysis using post-mortem prefrontal cortex data (GSE59286; *n* = 45) provided direct empirical support for this interpretation: the majority of blood candidate miRNAs showed no corresponding expression in brain tissue, with only hsa-miR-29c-5p demonstrating directional concordance across both tissues. These findings suggest that age stratification, platform harmonization, and cross-tissue validation should be considered essential prerequisites for reliable ASD miRNA biomarker discovery, rather than optional refinements.

**Supplementary Information:**

The online version contains supplementary material available at 10.1038/s41598-026-51487-x.

## Introduction

Biomarker discovery in autism spectrum disorder (ASD) faces a critical reproducibility challenge^1^. Despite numerous studies reporting differential expression of blood microRNAs (miRNAs) in ASD, cross-study validation remains elusive^2^. This lack of reproducibility mirrors broader concerns in biomarker research and raises fundamental questions about the reliability of reported findings.

MicroRNAs represent attractive biomarker candidates due to their stability in peripheral circulation, non-invasive accessibility, and established regulatory roles in neurodevelopment and gene expression^3^. However, published ASD miRNA studies frequently report non-overlapping sets of differentially expressed miRNAs, even when examining similar populations^4^. This inconsistency suggests that methodological factors, rather than biological differences, may drive observed heterogeneity. Members of several miRNA families, including the miR-29 and let-7 families, have been implicated in synaptic plasticity^5^ and neuronal differentiation^6,7^, respectively, suggesting biological plausibility for their involvement in neurodevelopmental conditions such as ASD.

Several sources of methodological heterogeneity have been identified in biomarker studies, including participant age, sample collection protocols, profiling platforms, and analytical approaches. Age represents a particularly underexplored factor, as miRNA expression patterns show dynamic changes across developmental stages^8^. Given that ASD studies typically examine broad age ranges spanning childhood through adulthood, age-related effects could substantially contribute to cross-study variability.

Meta-analysis provides a systematic approach to quantify heterogeneity and identify its sources. Recent reviews have highlighted the need for rigorous meta-analytical approaches in ASD biomarker research, emphasizing the importance of standardized protocols and validation frameworks^9^. However, no comprehensive meta-analysis has specifically examined the impact of methodological heterogeneity on ASD blood miRNA findings.

Here, we conduct the first systematic meta-analysis of ASD blood miRNA studies with explicit focus on methodological heterogeneity. Our objectives were to: (1) quantify between-study heterogeneity in miRNA expression patterns, (2) evaluate the impact of age and other methodological factors, (3) assess cross-validation stability, and (4) provide evidence-based recommendations for improving reproducibility. We hypothesized that methodological heterogeneity, including age-related and technical platform differences, may contribute to limited reproducibility of ASD blood miRNA signatures.

## Results

### Study characteristics and data overview

The systematic search of GEO database identified 24 potential datasets, of which 13 were blood-based studies. After screening for miRNA content and data availability, three eligible datasets were included: GSE89596, GSE67979, and GSE222046, comprising 90 participants (45 ASD, 45 controls) (Table 1). GSE89596 included adult participants (mean age 28.4 years) with peripheral blood samples profiled using Agilent microarray technology. GSE67979 and GSE222046 both focused on children (ages 2.5–7.5 years and 2–4 years, respectively) using peripheral blood samples, with GSE67979 employing Illumina MiSeq and GSE222046 using Illumina NextSeq platforms.

**Table 1:** Characteristics of included studies.

Study ID	Platform	Blood Matrix	Sample Size (ASD/control)	Age
GSE89596	GPL21575 (Agilent microarray)	Peripheral blood	30/30	28.4 yrs
GSE67979	GPL15520 (Illumina MiSeq)	Peripheral blood	5/5	2.5–7.5 yrs
GSE222046	GPL18573 (Illumina NextSeq)	Peripheral blood	10/10	2–4 yrs

After miRNA ID standardization and low-expression filtering, 614 miRNAs were included in the meta-analysis. Quality control assessment revealed normal hemolysis ratios across all samples. Principal component analysis confirmed successful batch effect correction, with no systematic clustering by study after ComBat normalization.

### Primary meta-analysis findings

No miRNAs achieved statistical significance after multiple testing correction (Benjamini-Hochberg FDR < 0.05 or < 0.10). However, seven miRNAs demonstrated exploratory evidence with unadjusted *p* < 0.01 and large effect sizes (|Hedges’ g| > 0.5) (Table 2).

**Table 2:** Top microRNA candidates with consistent evidence (*p* < 0.01, |g| > 0.5).

miRNA	Hedges’ g	95% CI	*P*-value	I² (%)
hsa-miR-29c-5p	0.653	0.228 to 1.077	0.003	0.0
hsa-miR-203b-3p	−0.652	−1.077 to −0.227	0.003	0.0
hsa-miR-2115-5p	0.623	0.200 to 1.046	0.004	0.0
hsa-miR-17-3p	−0.567	−0.990 to −0.143	0.009	0.0
hsa-miR-30c-1-3p	−0.563	−0.985 to − 0.141	0.009	0.0
hsa-miR-2116-5p	−0.561	−0.984 to −0.138	0.009	0.1
hsa-let-7f-5p	−0.557	−0.979 to −0.135	0.010	0.0

All candidates showed near-zero between-study heterogeneity (I² ≤ 0.1%), indicating consistent directionality across studies. Positive values indicate higher expression in ASD; negative values indicate lower expression in ASD. No miRNAs survived multiple testing correction (Benjamini-Hochberg FDR < 0.05). CI, confidence interval; g, Hedges’ g effect size; I², heterogeneity statistic.

**Fig. 1:**
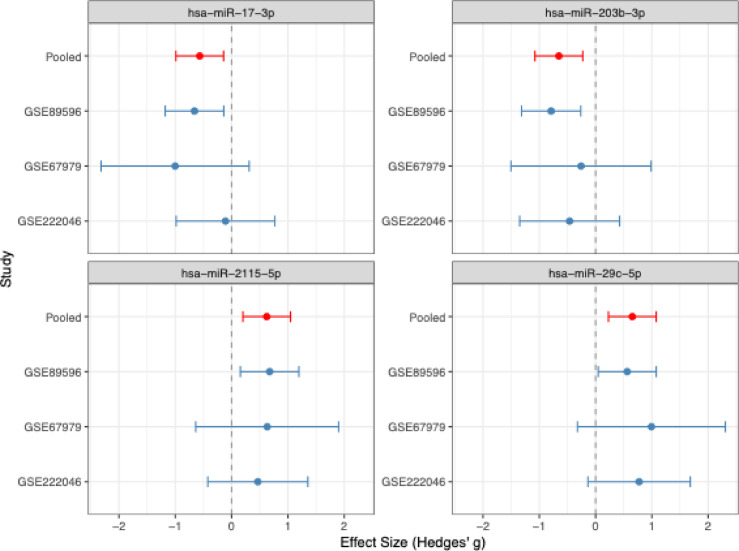
Forest plots for top miRNA candidates. Individual study and pooled effect sizes (Hedges’ g) with 95% confidence intervals for the seven miRNAs showing strongest evidence (*p* < 0.01, |g| > 0.5). All candidates demonstrated zero between-study heterogeneity (I² ≤ 0.1%), indicating consistent directionality across studies.

The top candidates were hsa-miR-29c-5p (g = 0.653, 95% CI: 0.228–1.077, *p* = 0.003), hsa-miR-203b-3p (g = −0.652, 95% CI: −1.077 to −0.227, *p* = 0.003), and hsa-miR-2115-5p (g = 0.623, 95% CI: 0.200–1.046.200.046, *p* = 0.004). Notably, all seven candidates showed near-zero between-study heterogeneity (I² ≤ 0.1%) and consistent confidence and prediction intervals, indicating consistent directionality across studies (Fig. 1).

At the broader significance level (*p* < 0.05), 44 miRNAs showed differential expression, though substantial heterogeneity was observed in many cases. The median I² across all 614 miRNAs was 28.7%, with 23% of miRNAs exhibiting I² > 50%.

### Heterogeneity patterns and extreme cases

Several miRNAs demonstrated extreme between-study variability. hsa-miR-423-5p showed the highest heterogeneity (I² = 89.2%, τ² = 1.89), followed by hsa-miR-320c (I² = 84.4%) and hsa-miR-486-5p (I² = 83.3%, τ² = 1.17). The prediction intervals for these miRNAs spanned large ranges, with hsa-miR-423-5p showing intervals from − 3.80 to 2.53, indicating massive uncertainty for future studies.

Influence analysis revealed that GSE222046 was an outlier for several miRNAs, most notably hsa-miR-486-5p (studentized residual = −3.38, Cook’s distance = 1.04). The analysis flagged this study as influential, demonstrating the substantial impact of individual studies on heterogeneity estimates in small meta-analyses.

### Age-related heterogeneity analysis

**Fig. 2:**
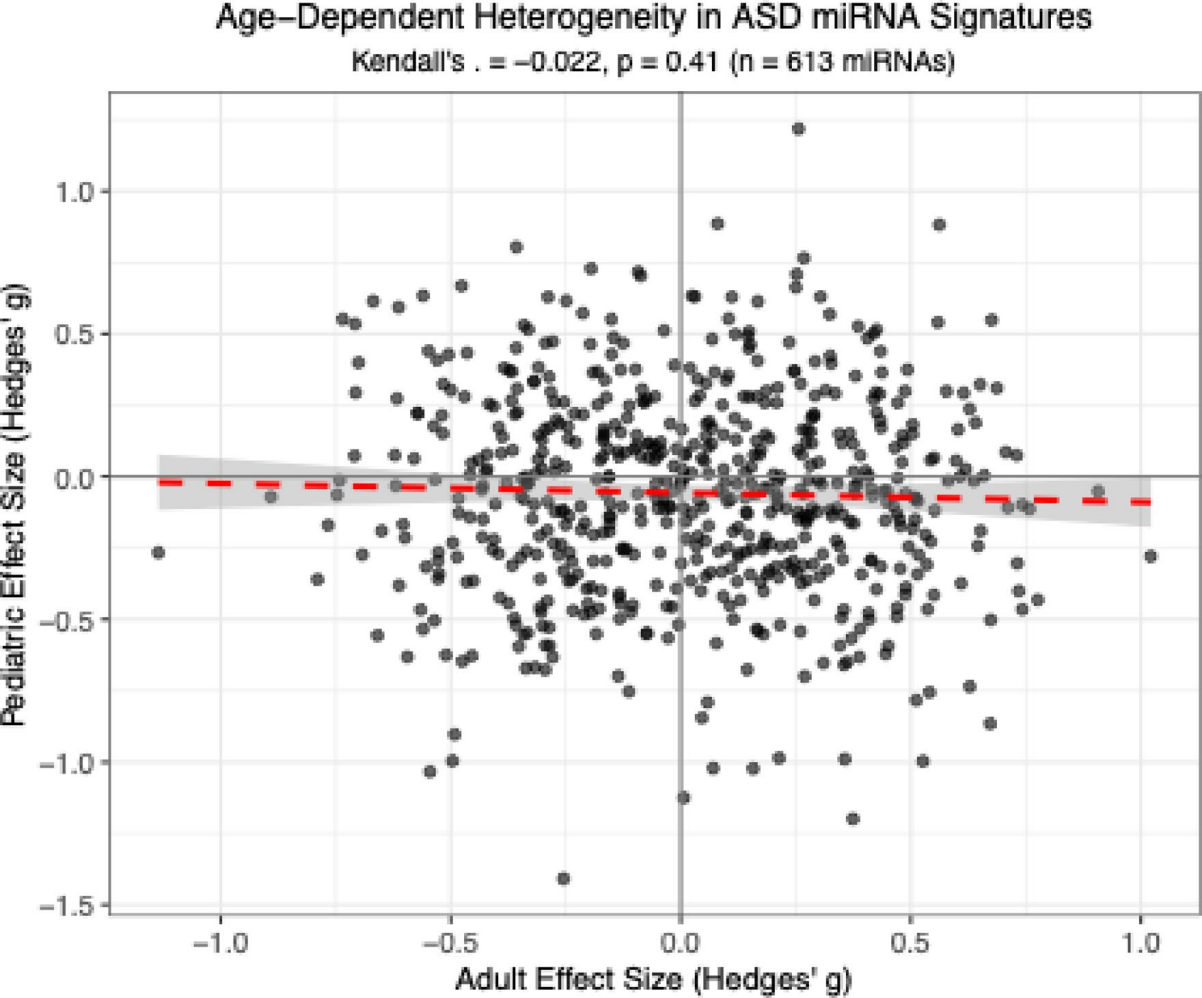
Age-dependent heterogeneity in ASD miRNA effect sizes. Scatter plot showing the correlation between adult (GSE89596) and pediatric (GSE67979, GSE222046) effect sizes for 614 miRNAs. Kendall’s τ = −0.022, *p* = 0.41, indicating near-zero correlation and possible differences in miRNA signatures across age groups, though this pattern may reflect age-related, platform-related, or combined effects.

Potential age-related and platform-related differences were observed throughout the dataset. The correlation between adult and pediatric effect sizes was near-zero (Kendall’s τ = −0.022, *p* = 0.410). It must be emphasized that this result is equally consistent with a technical artifact arising from the comparison of microarray-based profiling (GSE89596) with sequencing-based platforms (GSE67979, GSE222046) as it is with a genuine age-related biological difference, as age group and profiling platform are fully confounded in the available data. This near-zero correlation should therefore not be interpreted as evidence of age-specific miRNA signatures in ASD without independent replication in methodologically harmonized datasets (Fig. 2).

Meta-regression analysis identified borderline significant age effects for two miRNAs: hsa-miR-642a-3p (age coefficient β = −0.947, 95% CI: −1.876 to −0.018, *p* = 0.046) and hsa-miR-3127-5p (β = −0.895, 95% CI: −1.797 to 0.007, *p* = 0.052). These findings suggest that age modulates miRNA expression patterns, though limited power prevented detection of additional age-dependent effects.

### Cross-validation stability assessment

Leave-one-dataset-out (LODO) cross-validation revealed substantial instability when excluding different studies. Sign consistency (proportion of miRNAs maintaining directional consistency) ranged from 68.9% to 89.9% depending on which dataset was excluded.

Excluding GSE89596 (the adult cohort) resulted in the lowest sign consistency (68.9%), while excluding GSE67979 yielded the highest (89.9%). This pattern suggests that the adult dataset contributes different information compared to pediatric studies, though this difference may reflect age-related, platform-related, or combined effects.

Rank stability analysis showed limited overlap in top-ranked miRNAs across LODO iterations. When excluding GSE67979, 12 of the top 20 miRNAs remained consistent. However, excluding GSE89596 or GSE222046 resulted in only 8 and 7 overlapping miRNAs, respectively.

### Supplementary cross-tissue analysis

Cross-tissue analysis of blood candidate miRNAs in GSE59286 PFC tissue revealed limited correspondence between blood and brain expression patterns (Suppl. Fig. S1; Suppl. Table S3). Of the seven blood candidates, four could not be evaluated in brain tissue due to absence from the dataset (hsa-miR-203b-3p, hsa-miR-17-3p) or near-absent PFC expression (hsa-miR-2115-5p: 77.8% zero counts; hsa-miR-2116-5p: 82.2% zero counts; Suppl. Fig. S2). Among the three analysable candidates, only hsa-miR-29c-5p showed directional concordance across tissues (blood: g = + 0.653; brain: log2FC = + 0.596, *p* = 0.078), while hsa-miR-30c-1-3p and hsa-let-7f-5p demonstrated near-zero effect sizes in PFC despite significant blood signals. These findings provide direct empirical evidence that blood miRNA signals in ASD largely do not reflect PFC expression patterns, offering a biological basis for the limited clinical translation observed in prior biomarker studies. Among the seven candidates, hsa-miR-29c-5p was the only one to meet both the consistency criterion in the primary meta-analysis and directional concordance in brain tissue, and may therefore represent the highest-priority candidate for targeted follow-up.

## Discussion

Our exploratory meta-analysis suggests that age-related and methodological factors, including technical platform differences, may contribute to limited reproducibility in ASD blood miRNA research. The near-zero correlation between adult and pediatric effect sizes (τ = −0.022) is presented here as a methodological observation rather than a biological finding. This result is equally attributable to technical differences between microarray and sequencing platforms as to genuine age-related biological variation, and the two sources of variability cannot be disentangled in the current data. Future studies examining age-related effects in ASD miRNA research should ensure platform homogeneity to avoid this confound. The reduction in sign consistency from 89.9% to 68.9% when excluding the adult dataset demonstrates that age and platform homogeneity are critical, rather than merely optional, considerations for reproducible ASD miRNA findings.

Blood-based miRNA biomarkers represent a clinically accessible and non-invasive approach, but their relationship to central disease mechanisms has remained empirically unresolved at the individual candidate level. Prior studies have demonstrated that blood-brain miRNA correspondence is generally limited^10^, but it has remained unclear which specific candidates, if any, might bridge this gap. The current cross-tissue analysis addresses this directly: the majority of blood candidate miRNAs identified in the primary meta-analysis showed no corresponding differential expression in ASD PFC tissue, and two candidates were near-absent in brain tissue entirely. This result provides a candidate-level empirical basis for understanding why blood miRNA biomarker studies in ASD have failed to translate clinically, not merely because of methodological heterogeneity, but because the peripheral signals themselves may not index central pathophysiology.

The supplementary cross-tissue analysis provides further empirical support for the limited translational relevance of blood miRNA signals in ASD. Within this context of broad discordance, hsa-miR-29c-5p stands out as the only candidate to demonstrate directional concordance across both blood and brain PFC tissue, with upregulation in ASD observed in both contexts. While statistical significance was not reached in the brain dataset after correction, a finding that should be interpreted in light of the considerable methodological limitations of GSE59286, including its wide age range and absence of diagnostic metadata, the cross-tissue concordance itself provides a meaningful prioritization criterion that was not previously available. hsa-miR-29c-5p thus emerges as a data-driven priority target for validation in independent, age-stratified cohorts with matched brain and blood samples.

The observation that some miRNAs exhibit extreme heterogeneity (I² > 80%) while others show perfect consistency indicates that reproducibility challenges are not uniform across the miRNA landscape. The substantial influence of individual studies on heterogeneity estimates, particularly the ability of a single small study to shift I² from 83% to 0%, demonstrates the fragility of current evidence and the impact of limited sample sizes. Beyond age effects, platform differences (microarray vs. sequencing) showed modest associations with effect size variations. The single adult study (GSE89596) consistently showed the largest influence on heterogeneity estimates across multiple miRNAs, suggesting that differences between the adult and pediatric datasets, whether attributable to age, profiling platform, or both, represent a major source of methodological variability in the current data.

While seven miRNAs demonstrated consistent effects across studies with zero heterogeneity (I² ≤ 0.1%), the absence of statistical significance after multiple testing correction highlights the challenging landscape of high-dimensional biomarker discovery with limited sample sizes. The 614 miRNAs analyzed represent a substantial multiple testing burden that, combined with small total sample size (*n* = 90), creates an inherently conservative testing environment. Nevertheless, these seven exploratory candidate signals with *p* < 0.01 and consistent directionality across available datasets may warrant targeted follow-up in larger, age-stratified, and methodologically harmonized cohorts.

The inclusion of both adult and pediatric datasets in this study was not a deliberate design choice but a consequence of the limited availability of publicly accessible blood-based miRNA datasets in ASD research. Importantly, this cross-age comparison provided an opportunity to explore potential sources of between-study variability in an exploratory manner. While age-dependent variation in gene expression is well recognized, its impact on reproducibility in blood-based miRNA studies in ASD has not been systematically examined within a reproducibility-focused framework. Our analysis provides preliminary evidence highlighting this issue, and future studies should prioritize age-stratified designs to build on these observations.

Several limitations constrain our findings. The reliance on a single adult dataset prevents robust conclusions about age-specific effects, and small individual study sample sizes limit statistical power. The absence of intermediate age groups (6–16 years) represents a critical gap, while platform differences between microarray and sequencing add technical complexity. Furthermore, differences in sample preparation across datasets, including the use of exosome-derived miRNA fractions in one study, represent an additional source of technical heterogeneity that could not be fully accounted for in the current analysis. Given these constraints, all findings should be interpreted as exploratory and hypothesis-generating rather than confirmatory. The poor reproducibility of ranking patterns across leave-one-dataset-out iterations further underscores the instability of current evidence. Furthermore, with only three datasets included in the primary meta-analysis, consistency metrics such as sign consistency and near-zero I² values should be interpreted with appropriate caution, as false consistency is a known hazard at this scale. These metrics are best understood as hypothesis-generating signals rather than confirmatory evidence, and independent replication in larger, methodologically harmonized cohorts remains essential.

Based on our preliminary findings, we suggest that age stratification be considered an important priority in ASD miRNA research, with separate analysis for pediatric, adolescent, and adult populations. Minimum sample size requirements should be established based on realistic effect sizes (g = 0.3–0.7), and standardized protocols for sample collection and data processing should be adopted community-wide. The development of age-specific reference intervals and standardized analytical pipelines represent immediate priorities.

The vast genotypic and phenotypic heterogeneity of ASD poses fundamental challenges to biomarker discovery and has led to persistent controversies about universal biomarkers^11^. Our findings align with broader concerns about the “replication crisis” in ASD biomarker research^12^. While the identification of seven exploratory candidate signals suggests that reproducible signatures may exist, current evidence is insufficient to support clinical translation. This reflects broader challenges in the field, where despite numerous promising candidates across multiple domains, no ASD biomarker has achieved clinical acceptance^13^.

The present study contributes to this foundation primarily by identifying the methodological sources of irreproducibility in ASD blood miRNA research, with the supplementary cross-tissue analysis providing additional candidate-level evidence to guide future prioritization. Future efforts should focus on targeted validation in independent, age-stratified, and methodologically harmonized cohorts, with cross-tissue validation incorporated as a prioritization criterion where feasible. The establishment of such methodological foundations must precede biomarker application to ensure reliable translation to clinical practice.

## Methods

### Literature search and study selection

**Fig. 3:**
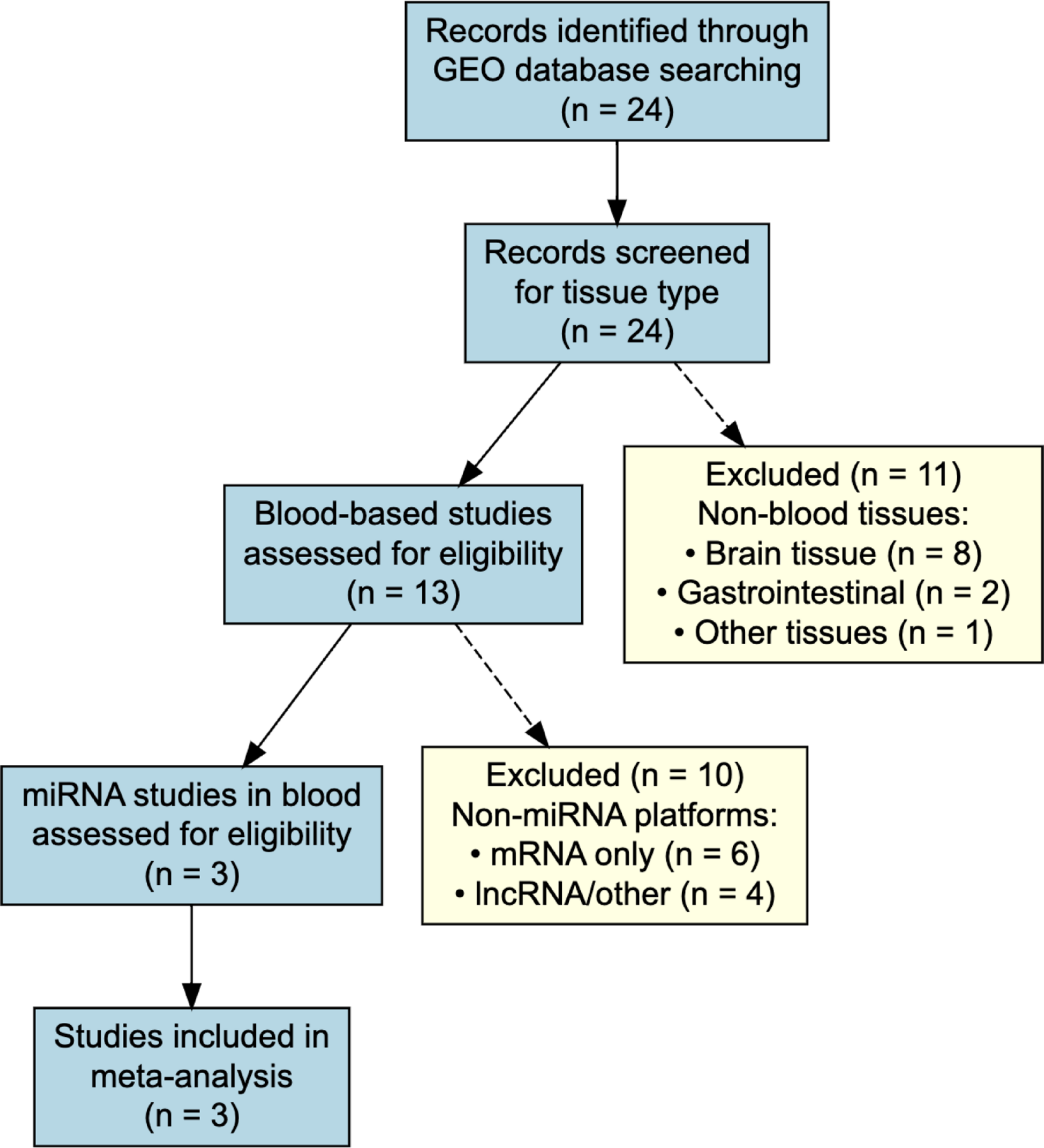
Study selection process for ASD blood miRNA meta-analysis. From 24 initial GEO database entries, 3 studies met inclusion criteria after screening for blood samples and miRNA content.

We conducted a systematic search of the Gene Expression Omnibus (GEO) database^14^, identifying 24 potential ASD miRNA studies. GEO was selected as the primary search resource as it represents the most comprehensive public repository for genome-wide expression data. In addition, ArrayExpress was searched for eligible datasets; however, no ASD-related datasets in ArrayExpress met our inclusion criteria for blood-derived samples with genome-wide miRNA profiling and available raw expression data. Brain tissue miRNA datasets identified during the search process were not eligible for the primary analysis but were retained for supplementary cross-tissue comparison (see below). After screening for blood-based samples, 13 studies remained. Of these, only 3 studies contained miRNA expression data with available raw data and appropriate controls (Fig. 3). Search terms included combinations of “autism,” “ASD,” “miRNA,” “microRNA,” “blood,” “serum,” and “plasma.” Studies were included if they met the following criteria: (1) human participants with ASD diagnosis and neurotypical controls, (2) blood-derived samples (whole blood, serum, or plasma), (3) genome-wide miRNA profiling data, and (4) publicly available raw expression data.

Studies were excluded if they: (1) lacked appropriate control groups, (2) focused exclusively on family members without ASD diagnosis, (3) used non-blood tissues, (4) provided only targeted miRNA analysis, or (5) lacked sufficient metadata for analysis. Two reviewers independently screened datasets, with disagreements resolved through discussion.

### Data extraction and processing

For each included study, we extracted participant characteristics (age, sex, diagnostic criteria), sample characteristics (blood matrix, collection protocol), and technical parameters (platform, preprocessing methods). Raw expression data were downloaded and processed using standardized protocols. Detailed participant characteristics, diagnostic criteria, and study design information for each included dataset are provided in Supplementary Table S1.

MicroRNA identifiers were standardized to miRBase v22 using miRBaseConverter to ensure consistent nomenclature across datasets^15^. Expression values were log2-transformed and quantile-normalized within each dataset. Common miRNAs across all studies were identified, and low-expression miRNAs were filtered using a 50% detection threshold (expressed in at least 50% of samples within each study).

Batch effects between studies were assessed using principal component analysis and corrected using ComBat when necessary^16^. Hemolysis contamination was evaluated using the miR-451a/miR-23a-3p ratio, with normal ranges considered 7–10^17^.

### Meta-analysis framework

For each miRNA, effect sizes were calculated as Hedges’ g with bias correction for small sample sizes. Random-effects meta-analysis was performed using the restricted maximum likelihood (REML) estimation method to account for between-study heterogeneity.

Between-study heterogeneity was quantified using I² statistics, τ² (tau-squared), and 95% prediction intervals^18^. I² values > 50% were considered indicative of substantial heterogeneity. Prediction intervals, which estimate the range of effects in future studies, were reported alongside confidence intervals.

### Multiple testing correction

Given the high-dimensional nature of miRNA data (614 miRNAs), multiple testing correction was applied using the Benjamini-Hochberg false discovery rate (FDR) procedure^19^. Statistical significance was evaluated at both unadjusted (*p* < 0.05, *p* < 0.01) and FDR-corrected (q < 0.05, q < 0.10) levels.

### Age-related heterogeneity analysis

To investigate potential age-related and platform-related differences, we performed subgroup analysis comparing adult (> 18 years) versus pediatric (≤ 18 years) populations. Meta-regression was conducted using age group as a moderator variable. It should be noted that age group and profiling platform were fully confounded in the available datasets, and therefore results of these analyses should be interpreted as exploratory observations rather than evidence of age-specific effects. The correlation between adult and pediatric effect sizes was assessed using Kendall’s tau correlation coefficient.

### Cross-validation and stability assessment

Leave-one-dataset-out (LODO) cross-validation was implemented to assess the stability of findings. For each iteration, one dataset was excluded and meta-analysis repeated on the remaining datasets. Sign consistency (proportion of miRNAs maintaining the same direction of effect) and rank consistency (overlap in top-ranked miRNAs) were calculated across LODO iterations.

### Influence analysis

Outlying studies and influential observations were identified using standardized residuals, Cook’s distance, and leave-one-out analysis. Baujat plots were generated to visualize the contribution of each study to overall heterogeneity.

### Supplementary cross-tissue analysis

To examine whether blood-derived candidate miRNAs reflect brain tissue expression, a supplementary analysis was performed using publicly available post-mortem prefrontal cortex (PFC) miRNA data from GSE59286 (ASD *n* = 20, Control *n* = 25; Illumina HiSeq 2000). Differential expression was assessed using DESeq2 with age included as a scaled continuous covariate to account for the wide developmental age range of the dataset (2 days to 61.5 years). Of the seven blood candidates, three were analysable in brain tissue after excluding candidates absent from the dataset (*n* = 2) or showing insufficient PFC expression (> 75% zero counts; *n* = 2). Full details of the cross-tissue analysis are provided in the Supplementary Methods and dataset characteristics are summarised in Supplementary Table S2.

### Software and reproducibility

All analyses were performed in R version 4.5.1 using the metafor package for meta-analysis^20^, sva package for batch correction, and additional packages for data processing and visualization. All analyses were conducted with set random seed (123) to ensure reproducibility. Code and processed data will be made available upon publication.

## Supplementary Information

Below is the link to the electronic supplementary material.

Supplementary Material 1

## Data Availability

All data generated in this study will be publicly released upon acceptance (archived on GitHub mirror provided). Data supporting the findings of this study are also available from the corresponding author, Dr. Seung-Nam Kim (snkim@dongguk.edu), upon reasonable request.
