# Supplementary material for: Potential contribution of age-related and methodological factors to limited reproducibility in autism spectrum disorder blood miRNA biomarker studies: an exploratory meta-analysis
Source: Sci Rep. 2026 May 2;16:20396. doi: 10.1038/s41598-026-51487-x (PMC13328733; doi:10.1038/s41598-026-51487-x)

Potential contribution of age-related and methodological factors to limited reproducibility in autism spectrum disorder blood miRNA biomarker studies: an exploratory meta-analysis

# Kwanghwan Lim^1,2^, Heejeong Shin^1^, and Seung-Nam Kim^1,*^

# ^1^College of Korean Medicine, Dongguk University, Goyang, South Korea.

# ^2^Bareun Kyunghee Korean Medicine Clinic, Seoul, South Korea.

# ^*^Correspondence: Seung-Nam Kim ([snkim@dongguk.edu](mailto:snkim@dongguk.edu))

# Supplementary Methods

# Brain Tissue Cross-tissue Analysis

## *Brain Tissue Dataset*

To investigate whether blood-derived candidate miRNAs reflect central nervous system pathology, we performed a supplementary cross-tissue analysis using a publicly available post-mortem brain miRNA dataset (GSE59286) obtained from the Gene Expression Omnibus (GEO) database. This dataset comprises miRNA expression profiles from prefrontal cortex (PFC) tissue of 20 ASD and 25 neurotypical control individuals, spanning a wide age range (2 days to 61.5 years). Raw count data were downloaded as supplementary files (GSE59286_exp_microRNA.txt.gz) and processed independently from the primary blood-based meta-analysis.

## *miRNA Identifier Mapping*

The brain dataset used an older miRBase nomenclature (lowercase, e.g., hsa-mir-29c) with minor strand annotations using asterisk notation (e.g., hsa-mir-30c-1*), whereas the blood meta-analysis used current miRBase annotations (e.g., hsa-miR-29c-5p). Manual mapping was performed based on strand identity: major strands (5p) were matched to the stem name, and minor strands (3p) were matched to the asterisk-annotated form. Of the seven blood candidate miRNAs, five could be mapped to the brain dataset; two (hsa-miR-203b-3p and hsa-miR-17-3p) were absent. Of the five mapped candidates, two (hsa-miR-2115-5p and hsa-miR-2116-5p) showed greater than 75% zero counts across brain samples and were excluded from differential expression analysis as insufficiently expressed in PFC tissue.

## *Differential Expression Analysis*

Raw count data were analysed using DESeq2 (v1.50.2) with a negative binomial generalised linear model. Low-expression miRNAs were filtered using a 50% detection threshold (expressed in at least 50% of samples). Age in days was included as a continuous covariate (scaled to zero mean and unit variance) in the design formula to account for the wide developmental age range of the dataset. Size factors were estimated using the median-of-ratios method. Differential expression between ASD and control groups was assessed using the Wald test. Results are reported as log2 fold change (log2FC) with standard error and unadjusted p-values. Given the exploratory nature of this analysis and the focus on directional concordance rather than statistical significance, adjusted p-values are reported for reference only.

Importantly, this brain tissue analysis carries several limitations. The dataset spans an exceptionally wide age range (2 days to 61.5 years for controls), and despite age correction, residual age-related confounding cannot be excluded. Furthermore, the dataset was not specifically designed for miRNA biomarker studies, and diagnostic criteria are not specified in the GEO metadata. These factors limit the interpretability of the cross-tissue comparison, and all findings should be regarded as exploratory.

**Supplementary Table S1.** Characteristics of included blood-based GEO datasets for the primary meta-analysis.

| **Study ID** | **Platform** | **Blood Matrix** | **Sample Preparation** | **Sample Size (ASD/Control)** | **Age** | **Sex (ASD; M/F)** | **Diagnostic Criteria** | **Comorbidity Information** |
| --- | --- | --- | --- | --- | --- | --- | --- | --- |
| **GSE89596** | GPL21575 (Agilent microarray) | Peripheral whole blood | Direct RNA extraction | 30 / 30 | Adults (mean 28.4 yrs) | 19M / 11F | DSM-5 + ADOS + SRS-2 | Not reported in metadata |
| **GSE67979** | GPL15520 (Illumina MiSeq) | Peripheral blood | Direct RNA extraction | 5 / 5 | Children (2.5–7 yrs) | 4M / 1F | DSM-IV-TR | Not reported in metadata |
| **GSE222046** | GPL18573 (Illumina NextSeq 500) | Peripheral blood | Exosome-derived miRNA (supercentrifugation) | 10 / 10 | Children (2–4 yrs) | Not reported | DSM-5 + ABC ≥67 + CARS ≥30 | Not reported in metadata |

Abbreviations: ASD, autism spectrum disorder; ADOS, Autism Diagnostic Observation Schedule; SRS-2, Social Responsiveness Scale, Second Edition; DSM, Diagnostic and Statistical Manual of Mental Disorders; ABC, Autism Behavior Checklist; CARS, Childhood Autism Rating Scale; M, male; F, female.

Note: GSE222046 miRNA expression data were derived from exosomes isolated from peripheral blood via supercentrifugation, which represents a distinct sample preparation method compared to direct RNA extraction used in the other two datasets. Comorbidity information was not available in the publicly accessible metadata for any of the three included datasets.

**Supplementary Table S2.** Characteristics of the brain tissue dataset (GSE59286) used for supplementary cross-tissue analysis.

| **Item** | **Description** |
| --- | --- |
| **GEO Accession** | GSE59286 |
| **Tissue** | Post-mortem brain tissue |
| **Brain Region** | Prefrontal Cortex (PFC) |
| **Platform** | Illumina HiSeq 2000 (GPL11154) |
| **Total Samples** | 45 |
| **ASD (n)** | 20 |
| **Control (n)** | 25 |
| **ASD Age Range** | 730–21,900 days (2.0–60.0 years) |
| **Control Age Range** | 2–22,452 days (0.005–61.5 years) |
| **Diagnostic Criteria** | Not specified in GEO metadata |
| **Comorbidity Information** | Not available in GEO metadata |
| **Total miRNAs** | 1,245 |
| **miRNAs after 50% expression filter** | 642 (full dataset) |
| **Differential Expression Method** | DESeq2 (negative binomial GLM) |
| **Age Covariate** | Continuous (age_days, scaled); included in design formula |
| **miRNAs analysed (blood candidate overlap)** | 3 of 7 blood candidates (hsa-miR-29c-5p, hsa-miR-30c-1-3p, hsa-let-7f-5p) |
| **miRNAs excluded (low brain expression)** | 2 of 7 (hsa-miR-2115-5p: 77.8% zero; hsa-miR-2116-5p: 82.2% zero) |
| **miRNAs absent from brain data** | 2 of 7 (hsa-miR-203b-3p, hsa-miR-17-3p) |

Abbreviations: ASD, autism spectrum disorder; PFC, prefrontal cortex; DESeq2, Differential Expression analysis using a Sequencing approach 2; GLM, generalised linear model.

Note: Diagnostic criteria and comorbidity information were not available in the publicly accessible GEO metadata for this dataset. The wide age range of the control group (2 days to 61.5 years) reflects the developmental span of the original study and represents a source of residual confounding despite age correction.

**Supplementary Table S3.** Cross-tissue comparison of blood candidate miRNAs: blood meta-analysis effect sizes versus brain PFC differential expression (GSE59286, age-adjusted).

| **miRNA** | **Blood Hedges' g** | **Blood 95% CI** | **Blood p-value** | **Brain log2FC (all)** | **Brain p-value (all)** | **Brain log2FC (Ped.)** | **Direction Concordance** |
| --- | --- | --- | --- | --- | --- | --- | --- |
| **hsa-miR-29c-5p** | +0.653 | 0.228 to 1.077 | 0.003 | +0.596 | 0.078 | +0.622 | Concordant |
| **hsa-miR-30c-1-3p** | -0.563 | -0.985 to -0.141 | 0.009 | -0.057 | 0.774 | +0.172 | Discordant |
| **hsa-let-7f-5p** | -0.557 | -0.979 to -0.135 | 0.010 | -0.086 | 0.638 | -0.068 | Concordant (weak) |
| **hsa-miR-203b-3p** | -0.652 | -1.077 to -0.227 | 0.003 | N/A | N/A | N/A | Not present in brain data |
| **hsa-miR-17-3p** | -0.567 | -0.990 to -0.143 | 0.009 | N/A | N/A | N/A | Not present in brain data |
| **hsa-miR-2115-5p** | +0.623 | 0.200 to 1.046 | 0.004 | Low expr. | — | — | Not analysable (77.8% zero) |
| **hsa-miR-2116-5p** | -0.561 | -0.984 to -0.138 | 0.009 | Low expr. | — | — | Not analysable (82.2% zero) |

Abbreviations: g, Hedges' g effect size; CI, confidence interval; log2FC, log2 fold change from DESeq2 (ASD vs. Control); Ped., Pediatric subgroup (≤18 years); N/A, not available (miRNA absent from brain dataset).

Note: Blood effect sizes and confidence intervals are derived from the primary random-effects meta-analysis. Brain log2FC values are from age-adjusted DESeq2 analysis of GSE59286 (full dataset, n=45). Direction concordance was primarily assessed based on the pediatric subgroup (≤18 years) log2FC where available, given that the primary blood meta-analysis included predominantly pediatric samples. Positive blood Hedges' g = higher expression in ASD; negative = lower expression in ASD.

**Supplementary Figure S1.** Cross-tissue comparison of ASD miRNA candidate signals. Forest plot showing effect sizes (with 95% confidence intervals) for three blood candidate miRNAs in blood (Hedges' g from meta-analysis, blue) and brain PFC (log2 fold change from age-adjusted DESeq2 analysis of GSE59286, red). Significance symbols: *, p < 0.05; †, p < 0.10; ns, p ≥ 0.10. Of the seven original blood candidates, three were analysable in brain tissue; the remaining four were either absent from the brain dataset (n=2) or showed insufficient expression in PFC (n=2; >75% zero counts). hsa-miR-29c-5p showed directional concordance between blood and brain (both upregulated in ASD), whereas hsa-miR-30c-1-3p and hsa-let-7f-5p showed near-zero effect sizes in brain despite significant blood signals, indicating that blood signals do not reliably reflect brain PFC expression patterns.


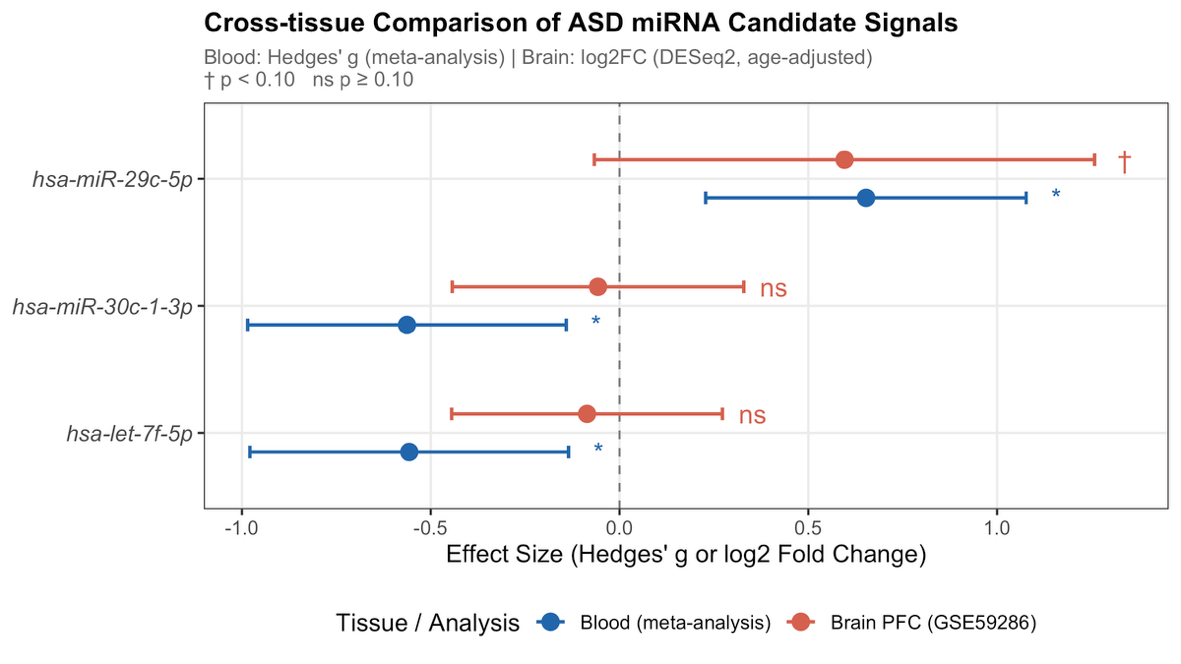


**Supplementary Figure S2.** Brain PFC expression levels of blood candidate miRNAs in ASD and control samples (GSE59286). Boxplots with individual data points showing log2(CPM+1) expression of five miRNAs mappable to the brain dataset across ASD (red) and control (blue) samples. The three left panels (white background) represent miRNAs with sufficient expression for differential analysis. The two right panels (grey background) represent miRNAs with >75% zero counts in brain tissue (hsa-miR-2115-5p and hsa-miR-2116-5p), which were excluded from the cross-tissue analysis. The near-absent expression of these candidates in PFC tissue suggests that their peripheral detection may reflect blood-specific biology rather than central nervous system pathology.


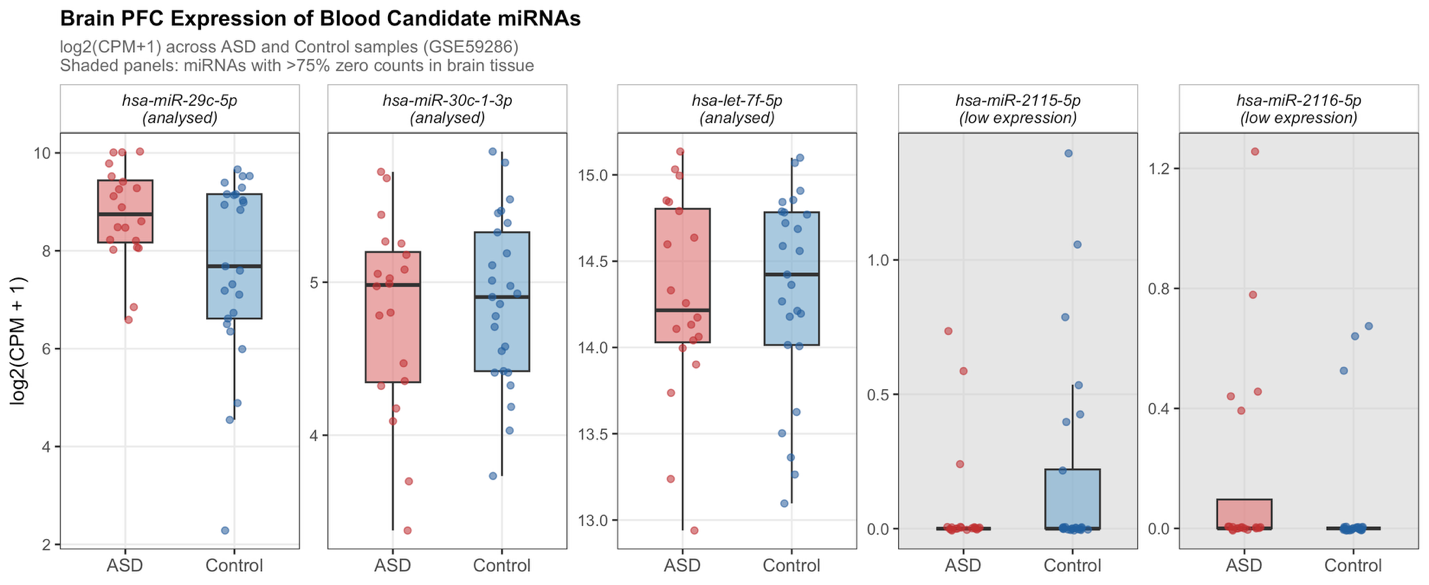

Supplement: Supplementary file 1 — Supplementary Material 1 [file 41598_2026_51487_MOESM1_ESM.docx]
